# Group A Streptococcus Bacteraemia Presenting as Acute Limb Ischaemia in a Neonate: A Case Report

**DOI:** 10.7759/cureus.80170

**Published:** 2025-03-06

**Authors:** Muhammad Moiz Muzaffar, Syahin Samani, Peter Van Der Velde

**Affiliations:** 1 Paediatrics, Worcestershire Acute Hospitals NHS Trust, Worcester, GBR

**Keywords:** group a streptococcus bacteraemia, group a β-haemolytic streptococci, invasive group a streptococcal infections, late onset sepsis, neonatal sepsis, neonate, septic emboli, septic embolism, thrombo-embolism

## Abstract

The reported incidence of Group A *Streptococcus* (GAS) bacteraemia is generally low, though it remains a significant concern. We report the case of a three-week-old neonate who presented with blackish discolouration of her toes and a rash, later diagnosed with invasive GAS septicaemia and septic emboli. She received antibiotics and anticoagulation, showing significant improvement, with mild residual hyperpigmentation and minor tissue loss at the two-month follow-up. Our case report highlights that early recognition and treatment of neonatal sepsis, including rare presentations such as septic emboli from pathogens like GAS, are critical for preventing severe complications and ensuring favourable long-term outcomes.

## Introduction

Neonatal infections can be broadly classified into early-onset sepsis (EOS) and late-onset sepsis (LOS). Group A *Streptococcus* (GAS) is a rare cause of infection in neonates, but it can lead to serious and potentially fatal infections in newborns [1]. In neonates, invasive GAS (iGAS) infection typically presents with rash, fever, and gastrointestinal disturbances [1]. We report a case of a neonate who initially had a blanching rash for two days and later developed discolouration in her right distal limb. Subsequently, it was discovered that she had an embolic occlusion, secondary to iGAS bacteraemia.

## Case presentation

A three-week-old neonate, born at term via vaginal delivery and with no significant medical history, presented to us with blackish discolouration of her toes. Two days before her presentation, she developed a rash around her face that spread all over her body.

**Figure 1:**
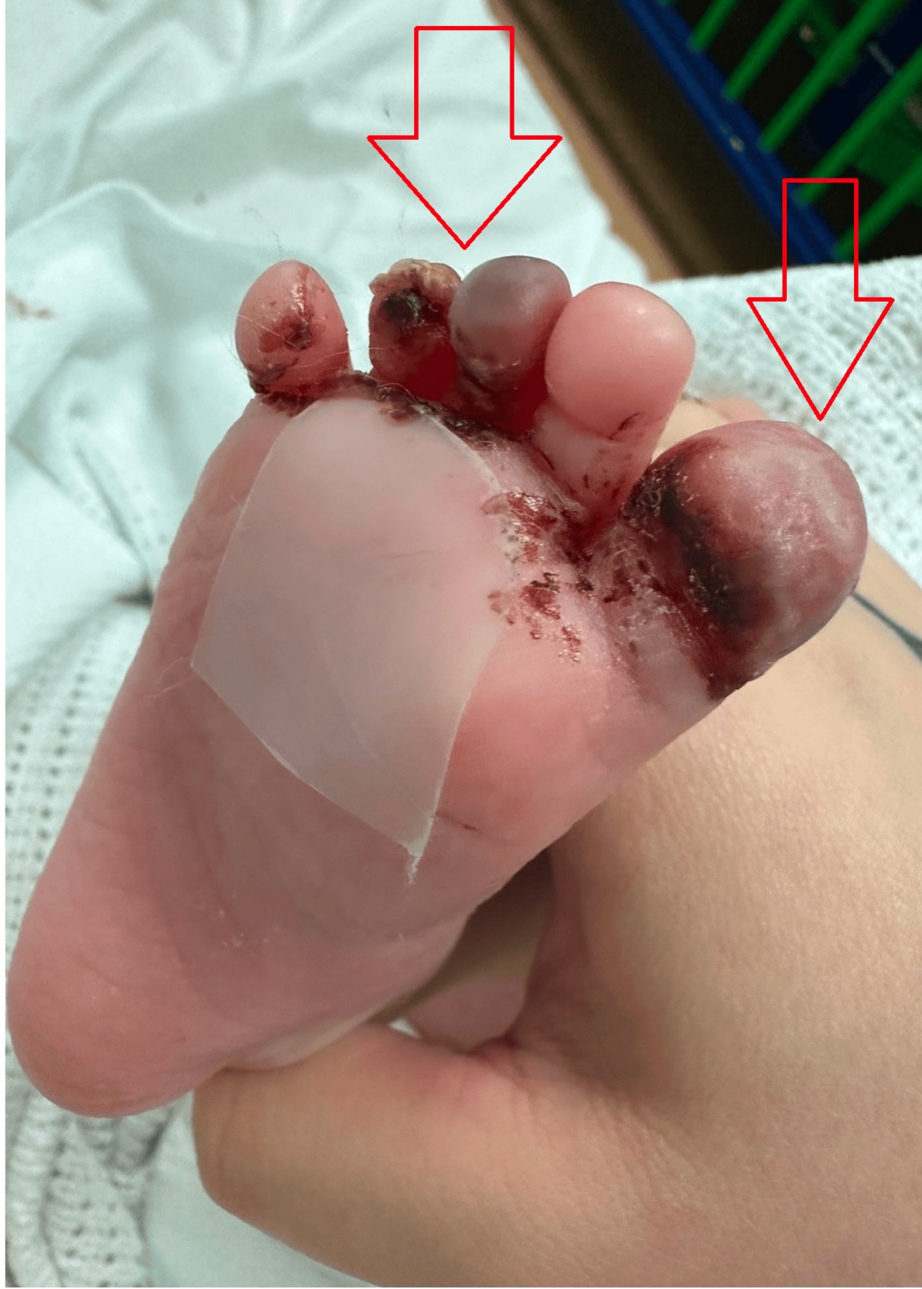
Red arrows showing the discoloured first, third, and fourth toes

On the initial presentation, she was unsettled, with tachycardia, tachypnea, and a raised temperature. She was noted to have a widespread blanching macular rash on the scalp, face, torso, and limbs, and a purpuric rash on the dorsum of her right foot. The first, third, and fourth toes appeared black/discoloured on the plantar aspect (Figure 1). 

She was initially managed for suspected meningococcal sepsis with intravenous (IV) fluid boluses and IV antibiotics. Though the rash and purpura were worrying, she turned out to be less systemically unwell than anticipated and responded quickly to the fluid boluses and antibiotics. The fever settled with antipyretics; however, the tachycardia did not.

There were no known infectious contacts, maternal risk factors for infection, or family history of clotting disorders, apart from maternal gestational thrombocytopenia.

She was started on IV cefotaxime and IV amoxicillin for suspected sepsis. Blood cultures confirmed GAS, and antibiotics were later changed according to microscopy, culture, and sensitivity (MC&S) to IV benzylpenicillin (14 days total) and oral clindamycin (five days). Her initial blood investigations are shown in Table 1.

**Table 1:** Initial investigation results WCC: White cell count; RCC: Red cell count; MCV: Mean corpuscular volume; MCH: Mean corpuscular haemoglobin; PT: Prothrombin time; INR: International normalized ratio; aPTT: Activated partial thromboplastin time; CRP: C-reactive protein; ALT: Alanine aminotransferase

Investigation	Value	Units	Range
Haemoglobin	110	g/L	115-165
WCC	18.4	109/L	5-19.5
Platelets	508	109/L	210-500
RCC	3.25	1012/L	3-5.4
MCV	101.2	fL	92-116
MCH	33.9	pg	30-36
Lymphocytes	3.1	109/L	2.5-16.5
Monocytes	0.7	109/L	0.7-1.5
PT	11	Seconds	9-14
INR	1.0	-	0.8-1.2
aPTT	27	Seconds	24-35
aPTT ratio	0.9	-	0.8-1.2
CRP	289	mg/L	0-5
Sodium	133	mmol/L	133-146
Potassium	5.1	mmol/L	3.5-5.3
Urea	3.6	mmol/L	0.8-5.5
Creatinine	24	umol/L	7-28
ALT	18	IU/L	6-30
Alkaline phosphatase	140	IU/L	113-443
Total bilirubin	14	umol/L	5-21
Globulin	30	g/L	18-36
Conjugated bilirubin	8	umol/L	0-3.4
Protein C activity	0.46	U/mL	0.20-0.64
Anti-thrombin activity	0.76	U/mL	0.41-0.93
Protein S activity	0.84	U/mL	0.22-0.78

The rest of the investigations included a throat swab culture that showed a heavy growth of candida and a mixed growth of bacteria. Her pneumococcal and meningococcal polymerase chain reaction (PCR) was negative. The urine culture showed no significant growth. Lumbar puncture was contraindicated, as the baby was anticoagulated.

**Figure 2:**
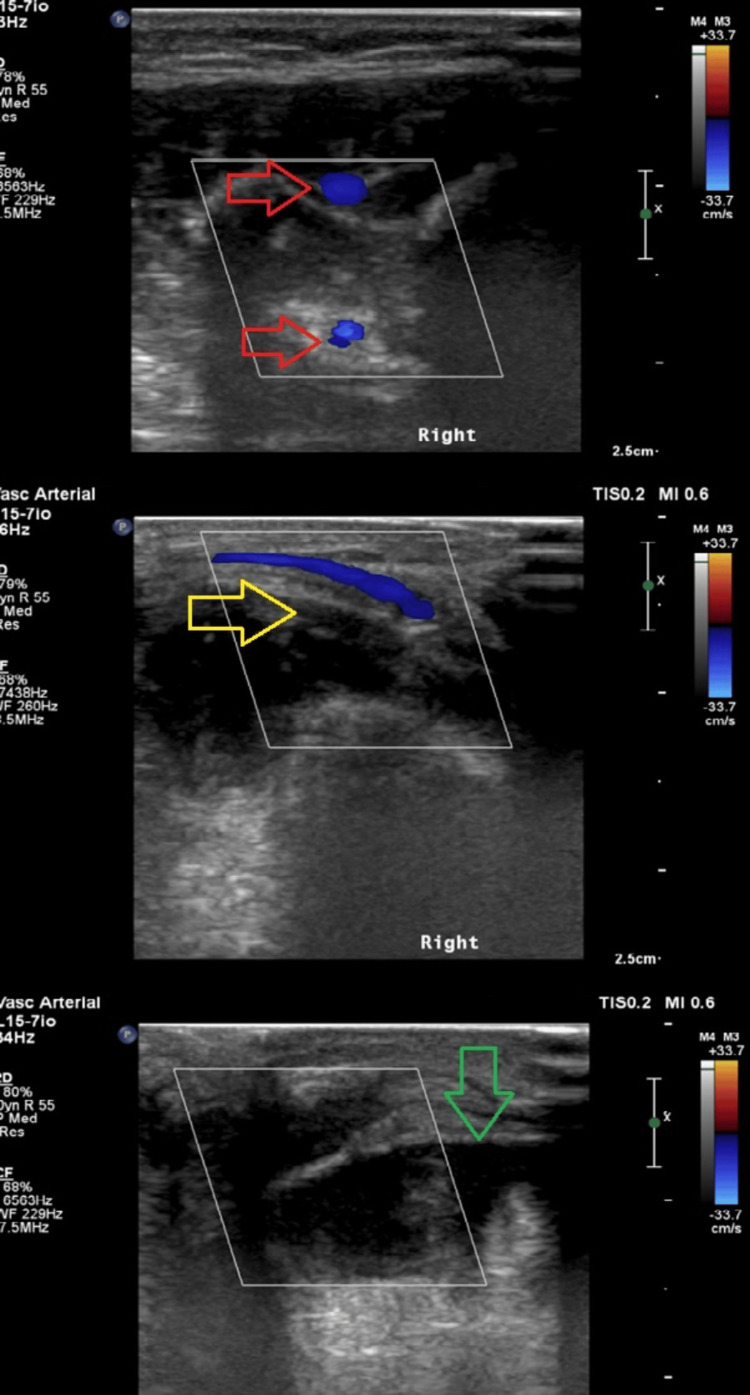
Doppler ultrasound images of the right lower limb Red arrows showing distal anterior tibial and peroneal artery; Yellow arrow showing posterior tibial artery over the medial malleolus; Green arrow showing absent signal in the dorsalis pedis artery.

**Figure 3:**
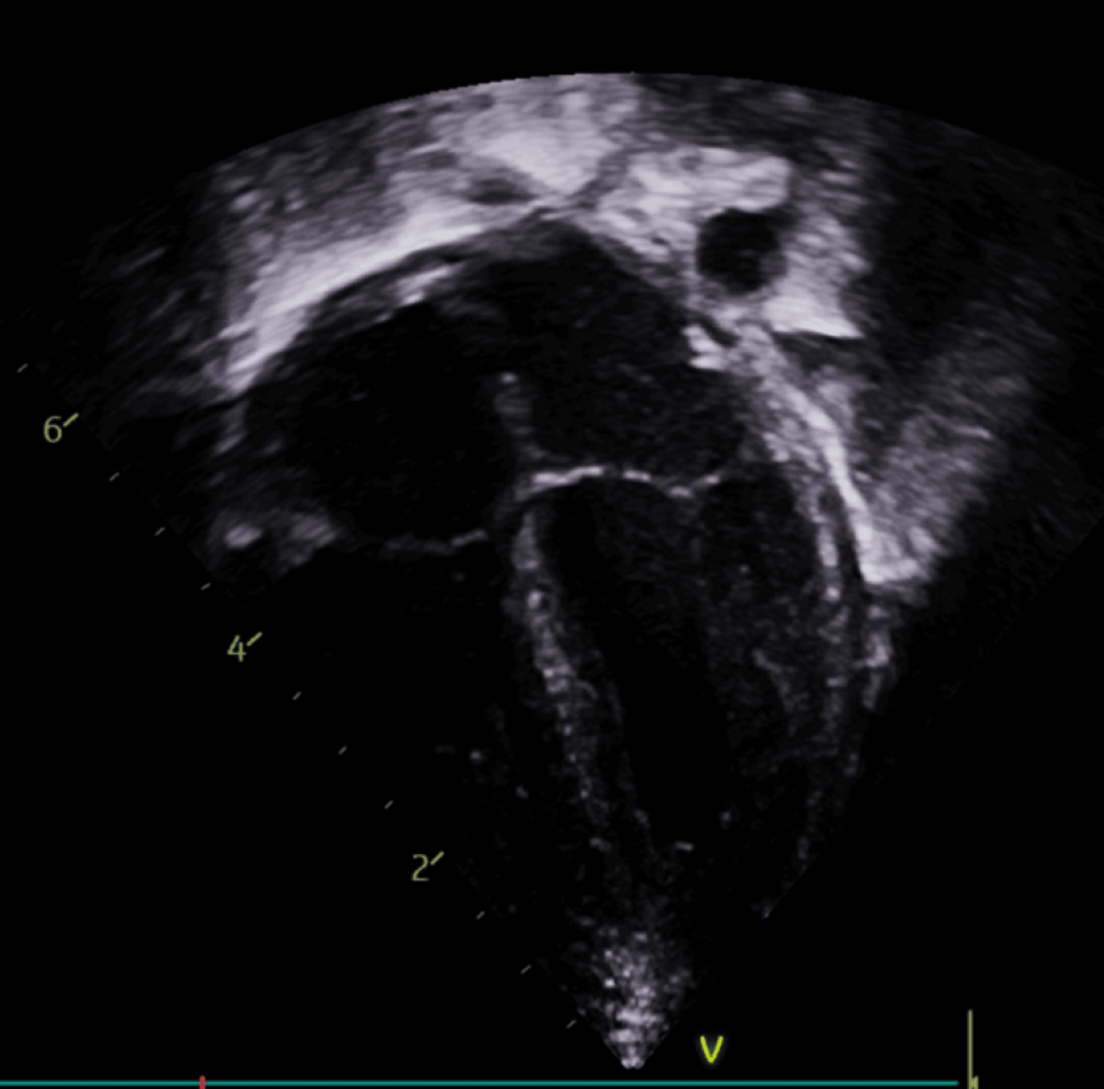
Echocardiogram image showing four chamber view of the heart revealing no abnormalities

**Figure 4:**
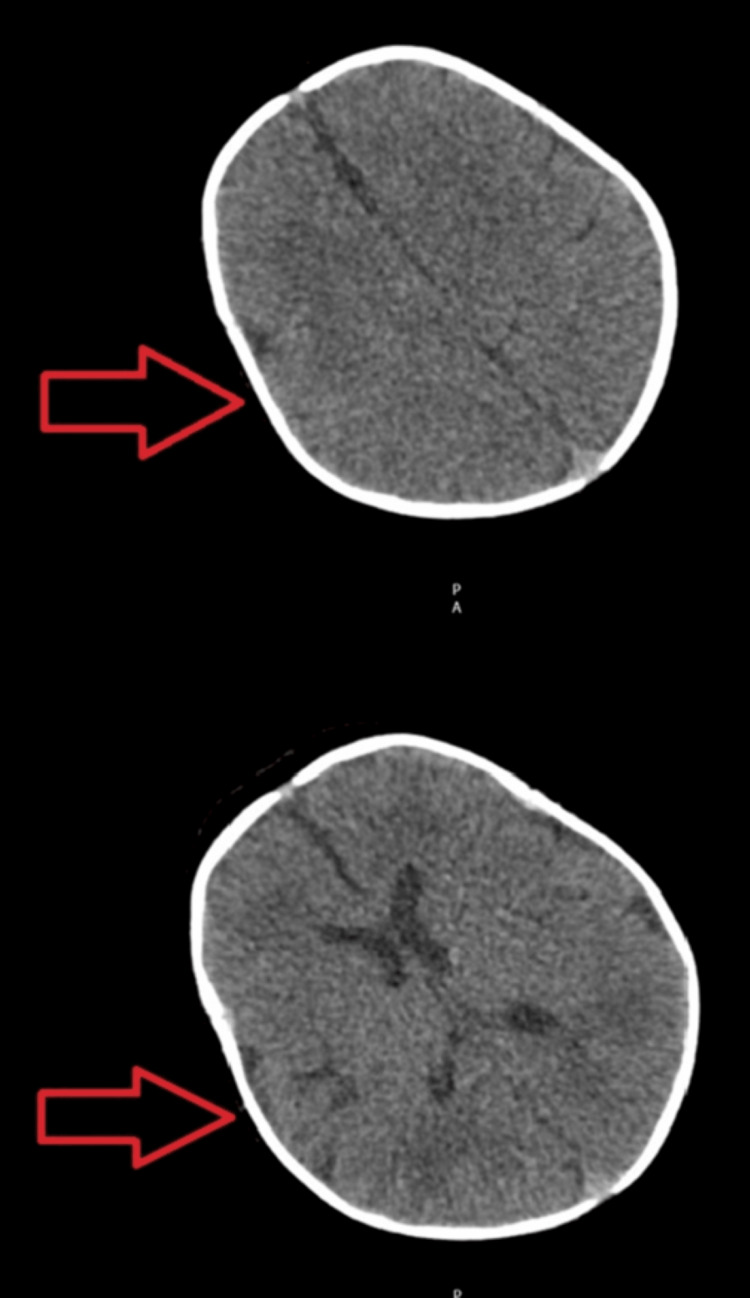
Arrows showing normal computed tomography of the head views

A Doppler ultrasound of the right lower limb was performed for her discoloured toes, suggesting embolic occlusion, with non-visualization of signal in the right dorsalis pedis artery (Figure 2). An echocardiogram (Figure 3) and computed tomography of the head (Figure 4) were done on the same day, which revealed no significant abnormalities. She was started on IV unfractionated heparin, which was later changed to low molecular weight heparin. Glyceryl trinitrate patches were also applied to the affected toes.

**Figure 5:**
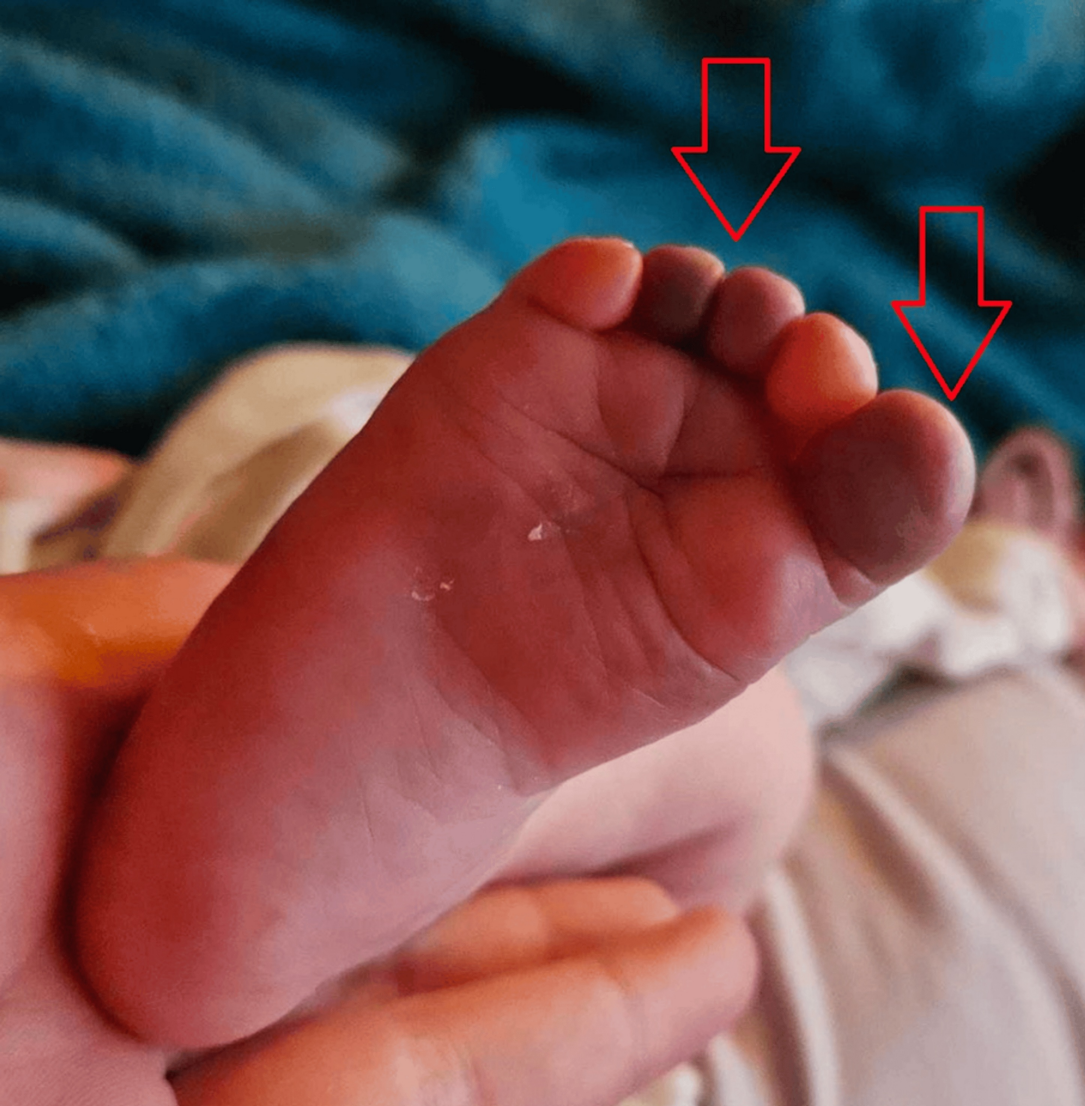
Arrows showing improvement in the first, third, and fourth toes.

She was treated for iGAS septicaemia with septic emboli to her toes and stayed as an inpatient for 21 days. She showed marked clinical improvement on the antibiotics, supported by a down-trending C-reactive protein (CRP) and improving observations. The Clexane was continued until there was no further improvement in the appearance of her toes, and the plastics team concluded that no surgical intervention was required (Figure 5).

She was reviewed in the clinic two months later and appeared systemically well. Her right foot had some slight hyperpigmentation over the dorsum, but all her toes looked pink and healthy, with only a tiny degree of tissue loss from the pulp of the fourth toe.

## Discussion

Neonatal sepsis presents as non-specific findings and thus requires a high degree of clinical suspicion. It is a clinical syndrome and relies on systemic signs and isolation of a pathogen from blood for diagnosis [2]. Neonatal sepsis is classified into EOS and LOS. EOS occurs within 72 hours after birth, while LOS is characterized by the onset of symptoms after 72 hours of life, due to exposure to microorganisms in the community or hospital environment. Pathogens range from gram-positive and harmful bacteria to viruses and fungi. Neonatal sepsis used to be primarily caused by GAS, but nowadays, GAS is rarely the cause. The World Health Organization (WHO) estimated a 0.55 neonatal GAS bacteraemia rate per 1000 live births in 2005 [3]. However, a recent meta-analysis found that neonatal iGAS incidence was 0.04 (95% CI: 0.03-0.05) per 1000 live births [4].

GAS is a highly contagious pathogen. It can colonize asymptomatic carriers or cause mild, localized, and self-limited infections like tonsillitis, scarlet fever, and impetigo. In some cases, it can lead to severe conditions such as toxic shock syndrome (TSS), necrotizing fasciitis (NF), and bacteraemia, which can then cause secondary localized infections, including meningitis, pneumonia, peritonitis, osteomyelitis, septic arthritis, myositis, surgical site infection, and postpartum sepsis.

Worldwide, more than one in five neonates die from infection secondary to iGAS, a case fatality rate that is comparable to, or possibly higher than, that reported for neonatal sepsis due to other infectious causes (11%-19%) [5], including group B streptococcal sepsis (8.4%; 95% CI: 6.6%-10.2%) [6], non-pneumonia and non-meningitis pneumococcal infection (31%; 95% CI: 13%-63%), and *Haemophilus influenzae* type b meningitis (19%; 95% CI: 7%-29%) [7].

Our case highlights how a seemingly healthy child with a rash had a severe underlying infection. The onset of discolouration on the toes was the only reason the child was brought to the hospital. On presentation, the newborn was not severely compromised, i.e., in disseminated intravascular coagulation (DIC) or septic shock, and only had subtle systemic signs along with the limb finding. The presence of septic emboli at presentation would typically occur in a severe infection or a critically unwell baby, possibly requiring ICU support throughout the diseased period, but this was not the case, and the baby showed a remarkable recovery [8]. 

Septic emboli in the neonatal population have not been extensively studied, and the medical literature is based only on case reports and case series. In neonates, distal limb ischaemia can be due to sepsis, coagulation disorders, iatrogenic injuries (catheterization of umbilical and peripheral arteries), dehydration, polycythaemia, and congenital nephrotic syndrome [9].

The diagnosis of limb ischaemia secondary to sepsis is primarily clinical. The sequential changes may include skin discolouration, sometimes combined with atrophy, ulceration, and necrosis. One non-invasive, widely accessible, low-cost imaging method that offers enough information to assess the condition and initiate appropriate treatment plans is ultrasound [10]. The goal is to reach the clinical decision in the limited time available and formulate a management plan as soon as possible to save the affected limb and avoid any long-term disability. 

The control of underlying infection with early antibiotic initiation and systemic stability constitutes the cornerstone of treatment. High-quality data for anticoagulation in the neonatal population is scarce, and although guidelines for management are available, they are primarily based on consensus recommendations and extrapolation from adult data. Anticoagulation therapy can prevent thrombus formation, potentially inhibit platelet function, and have an anti-inflammatory effect [11]. However, there is a risk associated with these consequences: they could spread infection and cause intracranial or systemic bleeding [11].

## Conclusions

Identifying neonatal sepsis can be challenging due to non-specific symptoms, necessitating a keen sense of clinical awareness for accurate diagnosis. Although GAS is becoming increasingly rare, it should still be considered a differential diagnosis in neonatal sepsis. The presence of septic emboli as a clinical sign in a haemodynamically stable baby is highly unusual. Our case highlights how the early start of antibiotics and prompt management of thrombo-embolic events can prevent long-term disability.
